# The HIM (Health for Izhevsk Men) trial protocol

**DOI:** 10.1186/1472-6963-8-69

**Published:** 2008-03-31

**Authors:** Susannah Tomkins, Elizabeth Allen, Olga Savenko, Jim McCambridge, Lyudmila Saburova, Nikolay Kiryanov, Alexey Oralov, Artyom Gil, David A Leon, Martin McKee, Diana Elbourne

**Affiliations:** 1 Department of Epidemiology and Population Health, London School of Hygiene and Tropical Medicine, London, UK; 2 Social Technologies Institute, Izhevsk, Russia; 3 Izhevsk Medical Academy, Izhevsk, Russia; 4 Izhevsk State Technical University, Izhevsk, Russia; 5 Department of Public Health and Policy, London School of Hygiene and Tropical Medicine, London, UK

## Abstract

**Background:**

Russia is one of the very few industrialised countries in the world where life expectancy has been declining. Alcohol has been implicated as a major contributor to the rapid fluctuations observed in male life expectancy since 1985 that have been particularly marked among working-age men.

One approach to reducing the alcohol problem in Russia is 'brief interventions' which seek to change views of the personal acceptability of excessive drinking and to encourage self-directed behaviour change. There is limited understanding in Russia of the salience and applicability of Motivational Interviewing (MI), a well-defined brief intervention commonly used to target alcohol-related behaviour, but MI may have important potential for success within the Russian context.

**Methods/Design:**

The study will be an individually randomised two-armed parallel group exploratory trial. The primary hypothesis is that a brief adaptation of MI will be effective in reducing self-reported hazardous drinking at 3 months. The secondary hypothesis is that it will be effective in reducing self-reported past week beverage alcohol consumption, alcohol dependence and related problems at 3 months and at 12 months. MI will also be effective at 12 months in reducing self-reported hazardous drinking, alcohol dependence and related problems, proxy reported hazardous drinking, and recent alcohol use as indicated by bio-markers.

Participants are drawn from the Izhevsk Family Study II, with eligibility determined based on proxy reports of hazardous drinking in the past year. All participants undergo a health check, with MI subsequently delivered to those in the intervention arm. Signed consent is obtained from those in the intervention arm at this point. Both groups are then invited for 3 and 12 month follow ups. The control group will not receive any additional intervention.

**Trial Registration:**

ISRCTN82405938

## Background

### What is the problem?

Russia is one of the very few industrialised countries in the world where life expectancy has been declining [1]. Analysis of routinely collected data over the past few decades has implicated alcohol as a major contributor to the rapid fluctuations observed in male life expectancy since 1985, that have been particularly marked among working-age men. For example, there was an increase in life expectancy following the start of the anti-alcohol campaign in 1985 [2] that led to falls in levels of per capita alcohol consumption in Russia [3-5] Rates of mortality from alcohol-related deaths among working age men decreased particularly sharply [5]. However, with the collapse of the Soviet Union this progress was reversed with a 6-year decline in male life expectancy between 1990 and 1994 that coincided with increases in per capita alcohol consumption. There is growing evidence linking excessive alcohol consumption to the high mortality among working age men in Russia [6,7]. A recent study demonstrated the large role played by alcohol, and in particular, of hazardous drinking patterns including a phenomenon termed "zapoi" (episodes of extended periods of drunkenness during which the participant withdraws from normal life), and consumption of surrogate alcohols (alcoholic substances not intended to be drunk, also known as non-beverage alcohols) in deaths from all causes and alcohol-related causes among men of working age in Russia [7].

### What is the evidence so far about what works to reduce alcohol problems?

One important approach to reducing the alcohol problem in Russia would be through government action to control manufacture and sale of drinkable surrogates and limit the availability and increase in price of legitimate alcoholic beverages. However there is also an urgent need to develop individual-level treatment services that are effective in the Russian context, where there has been little or no employment of individual counselling. There is an additional need to reach beyond specialist treatment services, as the majority of hazardous and harmful drinkers elsewhere do not access such provisions.

Another approach that may be appropriate is that of 'brief' interventions. These seek to change views of the personal acceptability of excessive drinking and to encourage self-directed behaviour change. They include both simple forms of advice and brief counselling techniques. Widespread dissemination of these interventions is possible as they can be delivered by a wide range of generic rather than specialist practitioners. These techniques have been shown to be effective in many other contexts. An international evidence-base has accumulated over more than 20 years, with efficacy data originating mainly, but not exclusively, from English language speaking countries. Reductions in volume of alcohol consumed are typically about 20–25% [8] and reductions in the proportions of hazardous drinkers are between 10–19% [9] The number needed to treat is about 8 for both hazardous alcohol consumption and for alcohol-related harm [9]. Reductions in alcohol problems of a similar magnitude and in health service utilisation have also been identified [10].

Motivational Interviewing (MI), a well-defined intervention [11], has been increasingly prominent within this literature in more recent years. One systematic review identifies 31 trials of MI, with alcohol being the most commonly targeted behaviour [12]. MI has been defined as "a facilitative, patient-centred counselling style for helping people explore and resolve ambivalence [11]." Brief adaptations of this counselling style offer promising potential to optimise the effectiveness of brief interventions. However, very little is known about the salience and applicability of these interventions in Russia – a country with a distinctive pattern of heavy drinking.

### Aims of the study

The study is a trial to explore the efficacy and acceptability of a brief intervention aimed at reducing the prevalence of hazardous drinking in working age men in a typical Russian city (Izhevsk). This will prepare the ground for subsequent effectiveness evaluations in a range of routine service settings with different populations of hazardous drinkers. Experience conducting this trial will provide an indication of the extent of possible benefit specifically within Russia. In addition, dissemination of information about the trial and its results is expected to contribute directly to improving understanding (locally, nationally and internationally) of the nature of this major problem in Russia and the required responses.

## Methods/Design

The study will be an individually randomised two-armed parallel group exploratory trial.

### Hypotheses

#### Primary hypothesis

A brief adaptation of MI will be effective in reducing self-reported hazardous drinking at 3 months.

#### Secondary hypotheses

MI will be effective in reducing self-reported past week beverage alcohol consumption, alcohol dependence and related problems at 3 months and at 12 months. MI will also be effective at 12 months in reducing self-reported hazardous drinking, alcohol dependence and related problems, proxy reported hazardous drinking, and recent alcohol use as indicated by bio-markers.

### Trial inclusion criteria

The men recruited into the trial will be drawn from a longitudinal observational study that is part of the Izhevsk Family Study II. This is based on 1750 men who were the controls in a case-control study of premature mortality conducted 2003–5 [7] supplemented by a further 250 men recruited using an identical protocol over a two month period in 2006. At initial recruitment to the case-control study, the men were aged 25–54 years and resident in Izhevsk. Interviews were conducted with 1750 control proxies, and 1691 of the controls themselves. For the supplementary group of 250 men, interviews were again carried out with proxies and with the men themselves. Interviewer-administered, structured questionnaires were used to gather information on a wide range of behaviours and characteristics, including alcohol consumption, tobacco use, a range of socio-economic and demographic factors, health status, and health-related behaviours.

Over an 18 month period (2007–8) as part of the longitudinal study we will attempt to recontact all of the 2000 men previously interviewed who are still alive and living in Izhevsk. Participants, who will be aged between 27 and 59 years of age, will be asked to take part in a re-interview study involving themselves as well as a proxy informant. Those who take part in this stage will then be invited to have a physical examination (referred to as a "Health Check") a few weeks later, to be carried out by a one of 4 specially trained doctors either in their own home or at a polyclinic in the city, according to the participant's preference.

Eligibility for entry to the trial will be determined according to information gathered at the initial re-interviews with proxy informants who live in the same households as the participants. These criteria are specified in Table 1. Men who had a proxy in the last study but now live alone, or for whom no proxy interview can be obtained, will be recruited on the basis of self reports of the same measures and using the same cut-offs.

**Table 1:** Eligibility and outcome definitions

Eligibility/Outcome	Criteria
Eligibility criteria for trial entry based on re-contact interview	*PROXY *reports (or self reports for men living alone) of hazardous drinking defined as one or more occurrences of any of the following: • zapoi in the past year • surrogates in the past year • hangover and/or excessive drunkenness and/or going to sleep clothed due to being drunk twice or more per week on average over the past year • weekly average of 250 mls or more of ethanol from beverages over the past year (ie 25+ UK alcohol units/week)

Primary outcome (3 months post-randomisation)	*SELF *report of hazardous drinking in defined as one or more occurrences of any of the following: • zapoi in the past month • surrogates in the past month • hangover and/or excessive drunkenness and/or going to sleep clothed due to being drunk twice or more per week on average over the past year • 250 mls or more of ethanol from beverages in the past week from beverages (ie 25+ UK alcohol units)

Secondary outcome (12 months post-randomisation)	*SELF *report of hazardous drinking defined as one or more occurrences of: • *as above at 3 months*

Supplementary secondary outcome (12 months post-randomisation)	*PROXY *report of hazardous drinking defined as one or more occurrences of: • *as above at 3 months except proxy rather than self- report* *SELF *completed: • AUDIT questionnaire (W.H.O. developed global screening assessment of hazardous drinking [26] • Leeds Dependency questionnaire [27] • SIP (Short Index of Problems) questionnaire [28] Biomarkers of: • liver damage (GGT, ALT, AST) • recent heavy drinking (CDT) [29]

### Trial exclusion criteria

Refusal to have a baseline health check and/or refusal to be followed up at 3 and 12 months will result in exclusion from the trial.

### Randomisation and consent

Although in most trials, consent to random allocation is sought before the allocation takes place, there are some acceptable exceptions when randomisation is carried out prior to consent (Zelen randomisation), as in this trial (see Ethics, below).

Data collected at the baseline interview will be sent to the randomisation service in London, allowing participants to be allocated to intervention or control prior to their health check appointment. Minimisation criteria (including age, surrogate use in past year, and living alone status) will be used to ensure a reasonable balance with respect to the principal known confounding factors.

Signed consent for taking part in the active arm of the trial will be obtained at the end of the health check. This is the last of a sequence of consents that the participant will need to give in the course of recontact as outlined in Table 2. Consent will be obtained differently for the intervention and control groups:

**Table 2:** Consent

What	When	Who obtains consent?	Comments
Oral consent for interview	Baseline recontact interview	Interviewer	Recorded by interviewer in questionnaire

Oral consent to be recontacted in future to assist with research (not-specific)	Baseline recontact interview	Interviewer	Recorded by interviewer in questionnaire

Expression of interest in health check following brief description of health check- copy of which to be left with participants	Baseline recontact interview	Interviewer	NOT consent per se, but indication of whether interested in taking part and if so, contact details (telephone number)

Signed consent for health check procedures	Health check	Doctor	Participants given information on BP and anthropometry and questionnaire, and blood taking

Signed consent to take part in active intervention arm of MI trial	Health check	Doctor	For subset of participants who are eligible, randomized to intervention arm of trial, and turn up for health check. Note, no explicit consent for being in control arm, beyond oral recontact agreement

a) Intervention group: Consent will be sought at the time of the health check by medical personnel involved. They will be trained to explain the purpose of the trial, provide written information and answer "frequently asked questions" about the trial. The consent being requested is (i) to take part in the intervention and (ii) to provide follow up data in 3 and 12 months time for the longitudinal study (even if they are not willing to take part in the intervention);

b) Control group: Consent to take part in the trial will not be sought from men randomised into the control group as they need to remain unaware of the trial (see Ethics, below). However, they will have previously given general consent to be followed up at the time of the initial re-interview.

### Interventions

#### a) Treatment group

MI will be delivered following completion of the health check (part of the longitudinal study protocol). An adaptation of MI has been developed for the Russian context, and includes topics such as surrogate drinking and zapoi. MI involves empathic questioning and listening, with a view to the development of discrepancy between alcohol use and other goals and values and the eliciting of personalised statements about change. The full intervention comprises up to four sessions. These will be delivered at home or in a clinic by specially trained practitioners with the two core sessions being approximately two weeks apart, with additional sessions (up to a maximum of 2) being available upon request. Practitioners have been trained and supervised in Russian, with a period of practice-based learning following an introductory workshop. Approximately 10% of sessions will be audio-recorded for quality control and supervision purposes.

#### b) Control group

This group will not receive any intervention other than having a health check as part of the longitudinal study and being invited for 3 and 12 month follow ups.

### Outcome measures

Follow-up data collection for both groups will take place after 3 and 12 months

Outcome interviews at 3 and 12 months will be partly interviewer-administered, and partly self-completed by participants, in order to replicate the administering of the same tools at baseline. The primary and secondary outcomes at 3 and 12 months are summarised in Table 1.

### Recruitment rate

Assuming that the overall prevalence of hazardous drinking remains the same over time, we have estimated from the original Izhevsk Family study data that approximately 25% of the 2000 men initially invited to take part in the Izhevsk family study II will be eligible for inclusion in the trial. Assuming a 60% response rate from those invited to participate in the Izhevsk family study 2 we would expect to be able to identify 300 hazardous drinkers eligible for the trial.

### Sample size

Based on current MI literature it is expected that 25% of participants in the intervention arm will stop hazardous drinking, as those in the control arm will have had minimal contact with the study and will not have been specifically told they are involved in research relating to alcohol, spontaneous reduction in hazardous drinking of more than 5% is unlikely. We have selected a difference at approximately the upper limit of the projected effect size based on previous meta-analyses [8] as a result of this unusual characteristic of the study design. We are unaware that such a Zelen design (see later) has previously been used in any study of brief alcohol intervention. Power calculations are therefore based on detecting a 20% difference between randomised groups (from 95% to 75%) with 90% power at the 5% level of statistical significance. A sample of at least 130 men (65 in each arm) will therefore be required. As randomisation was carried out prior to consent to MI, we assume that 20% of the participants in the intervention arm will not agree to receive the intervention. We also assume that there will be a 20% loss to follow for the 3 month assessment in both trial arms. This requires inflating the sample size to between 200 and 250 participants.

It should be noted that one aim of this exploratory trial will be to assess the assumptions behind these calculations in order to design a definitive study with power to assess smaller differences which could have nonetheless have major public health benefits.

### Type of analysis

The primary analysis will be based on a difference in the number of men classified as hazardous drinkers at the 3 month assessment between the randomised groups using the intention to treat principle. This may dilute the treatment effect but the analysis will not be biased if the loss to follow up, especially for the primary outcome is similar between the two randomised groups.

Differential effects by subgroup analyses based on important prognostic factors such as age group and severity of alcohol dependence will be assessed using interaction tests. We are aware that in this exploratory trial the results of such analyses will only be indicative.

In addition, the availability of the baseline information on all participants in the Izhevsk family study II will enable us to assess the extent to which those people included in the trial differ systematically from the population sample from which they were drawn, and will provide external validation for any trial findings.

### Frequency of analysis

An independent Data Monitoring Committee (DMC) will review, in strict confidence, data for the 3 month outcomes from the trial approximately 12 months from the start of the recruitment period. The Chair of the DMC may also request additional meeting/analyses. In the light of these data and other evidence from relevant studies, the DMC will inform the Trial Steering Committee (TSC) if in their view:

i. There is proof that the data indicate that any part of the protocol under investigation is either clearly indicated or clearly contra-indicated either for all patients or a particular subgroup of patients.

ii. It is evident that no clear outcome will be obtained with the current trial design.

iii. That they have a major ethical or safety concern.

### Publication policy

To safeguard the integrity of the trial, data from this study will not be presented in public or submitted for publication without requesting comments and receiving agreement from the Trial Steering Committee. The primary results of the trial will be presented first to the trial local investigators, and will be published by the group as a whole with local investigators acknowledged. The requirements for authorship will follow recommended practice in journal guidelines. A summary of the results of the trial will be made available on the trial website.

### Ethics and confidentiality

In the light of current research, it is not known whether MI will reduce the extent of hazardous drinking in this context. Hence randomisation as a means of allocating individual to be offered or not to be offered MI is the most ethical approach. Recent work [13] has shown that drinking behaviour can substantially change in response to participants' knowledge of being part of an alcohol-related research project. If we were to follow common randomised controlled trial practice, we would tell all potential participants about the trial including the alcohol-specific MI intervention and the alcohol-specific outcomes, and then ask for consent to be randomised to either arm of the trial. This approach would both create an 'artificial' control group sensitised to alcohol-related research which did not reflect the current experience of hazardous drinkers in Izhevsk, and could also seriously dilute any effect of the MI intervention. In this trial, therefore, we are restricting contact with the control group (especially in relation to being in a trial concentrating on alcohol) by using a Zelen single consent trial [14,15] in which the control group is only asked for their consent to follow up in health-related research, whereas the intervention group is also asked for consent to the MI. This design is ethically acceptable when the benefit of the intervention is unknown but when detailed knowledge of the trial and its exact purposes are likely to significantly bias or influence results [16-25].

Data about patients will be identified by their trial number to ensure confidentiality.

## Competing interests

The author(s) declare that they have no competing interests.

## Authors' contributions

DL had the original idea for the study. DL, JM and DE developed the design of the study to obtain funding. All authors contributed to the refinement of the protocol for ethical approval, read and approved the final manuscript. ST and EA prepared the final draft for publication.

## Pre-publication history

The pre-publication history for this paper can be accessed here:


